# Author Correction: An inorganic-rich but LiF-free interphase for fast charging and long cycle life lithium metal batteries

**DOI:** 10.1038/s41467-024-53498-6

**Published:** 2024-10-24

**Authors:** Muhammad Mominur Rahman, Sha Tan, Yang Yang, Hui Zhong, Sanjit Ghose, Iradwikanari Waluyo, Adrian Hunt, Lu Ma, Xiao-Qing Yang, Enyuan Hu

**Affiliations:** 1 Chemistry division, Brookhaven National Laboratory, Upton, NY 11973 USA; 2 National Synchrotron Lightsource II, Brookhaven National Laboratory, Upton, NY 11973 USA; 3 Department of Joint Photon Sciences Institute, Stony Brook University, Stony Brook, NY 11970 USA

Correction to: *Nature Communications* 10.1038/s41467-023-44282-z, published online 18 December 2023

In Fig. 1b the line colour labels in the legend were inadvertently flipped. The original legend labelled the Li metal data as orange and the NMC811 data as blue. The corrected legend labels the Li metal data as blue and the NMC811 data as orange.

The original article has been corrected.

