# Supplementary material for: An inorganic-rich but LiF-free interphase for fast charging and long cycle life lithium metal batteries
Source: Nat Commun. 2023 Dec 18;14:8414. doi: 10.1038/s41467-023-44282-z (PMC10728193; doi:10.1038/s41467-023-44282-z)
Supplement: Supplementary file 1 — Supplementary Information [file 41467_2023_44282_MOESM1_ESM.pdf]

Supplementary information for

An inorganic-rich but LiF-free interphase for fast charging and long cycle life lithium metal batteries

Muhammad Mominur Rahman,[1] Sha Tan,[1] Yang Yang,[2] Hui Zhong,[3] Sanjit Ghose,[2] Iradwikanari Waluyo,[2] Adrian Hunt,[2] Lu Ma,[2] Xiao-Qing Yang,[1] and Enyuan Hu[1*]

1. Chemistry division, Brookhaven National Laboratory, Upton, NY 11973, USA
2. National Synchrotron Lightsource II, Brookhaven National Laboratory, Upton, NY 11973, USA
3. Department of Joint Photon Sciences Institute, Stony Brook University, Stony Brook, NY, 11970

Email: enhu@bnl.gov

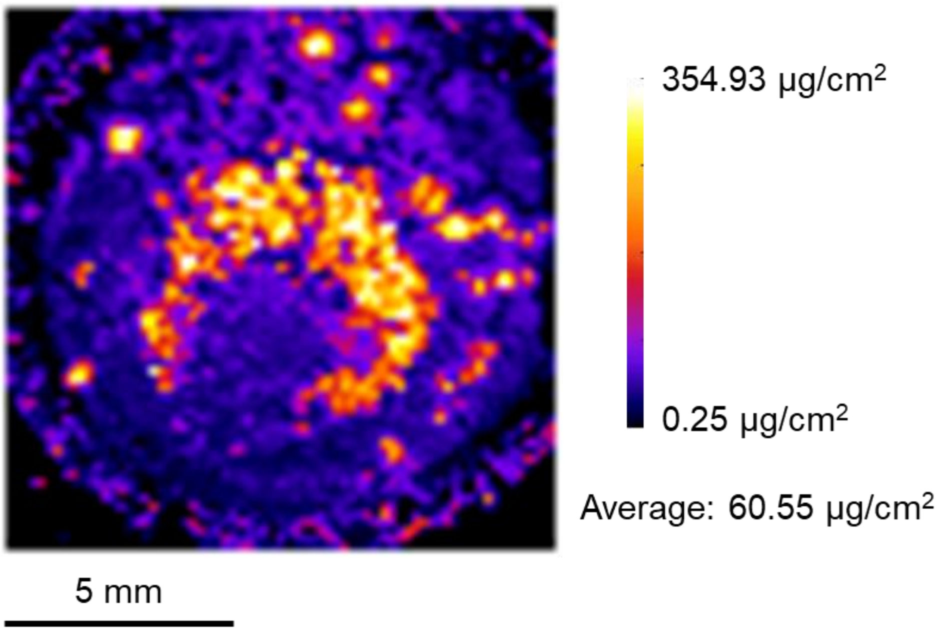

Figure S1. X-ray fluorescence map of Cs distribution on Li metal after 50 cycles. The cycling was performed at C/2 rate (0.8 mA/cm^2).

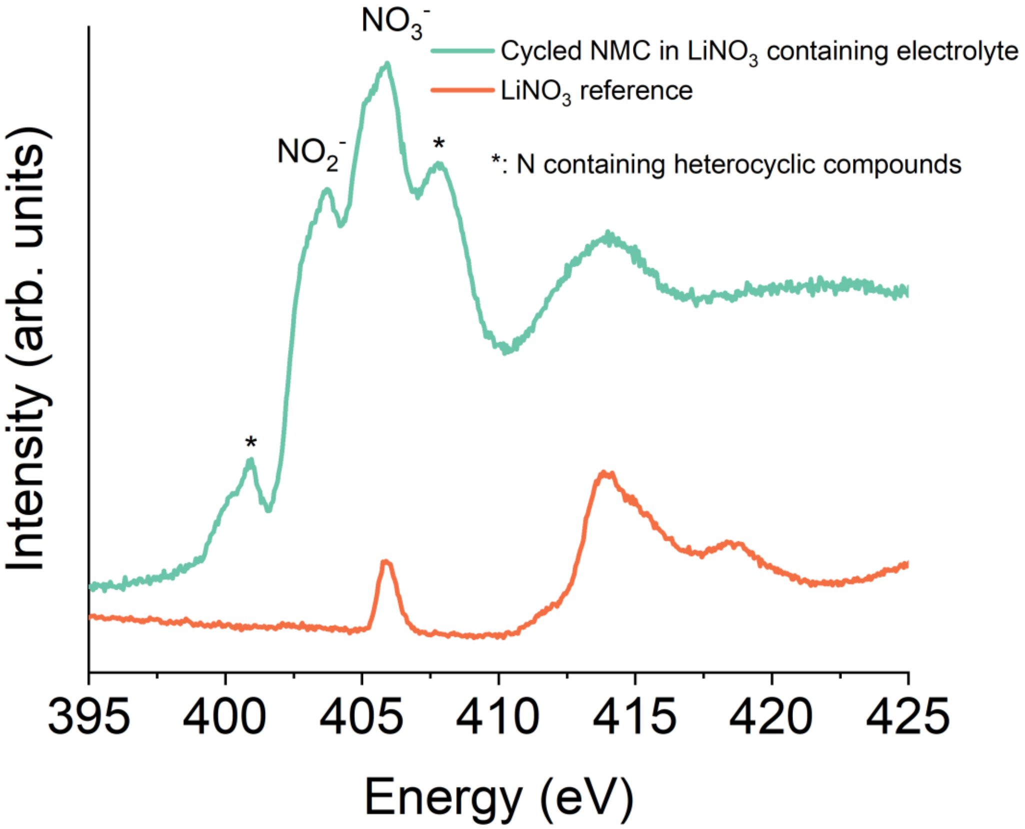

Figure S2. N K-edge of the cycled NMC811 surface in $LiNO_3$ additive containing electrolyte after 200 cycles.

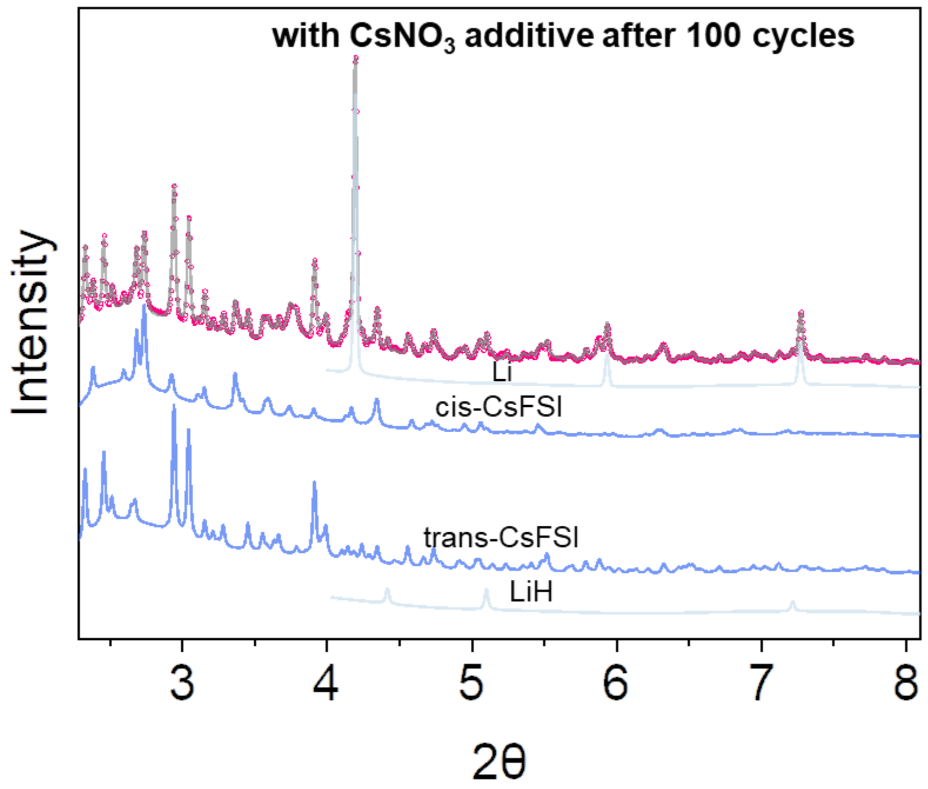

Figure S3. Rietveld refinement of the SEI species of Li metal cycled in the $CsNO_3$ containing electrolyte after 100 cycles.

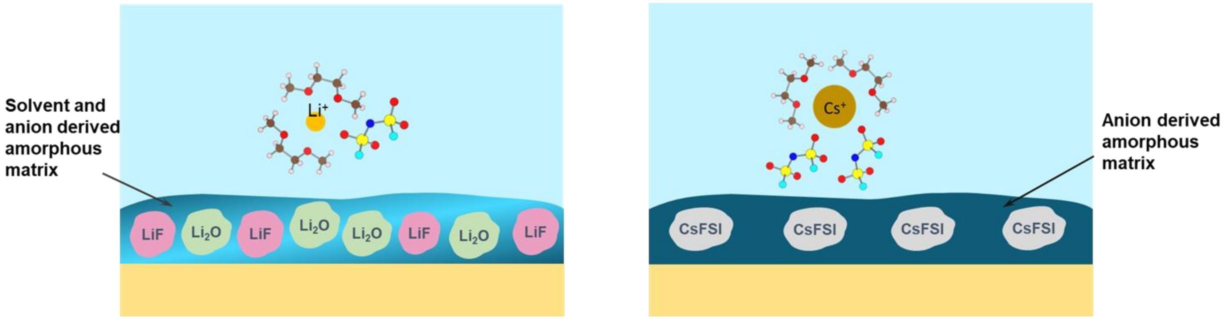

Figure S4. Illustration of the SEI formation mechanism. Light blue color indicates bulk electrolyte and dark blue color indicates the amorphous matrix of the SEI in the illustration. Color code for the atoms: red: oxygen; blue: nitrogen; cyan: fluorine; brown: carbon; white: hydrogen; gold: lithium; dark yellow: cesium, light yellow: sulfur.

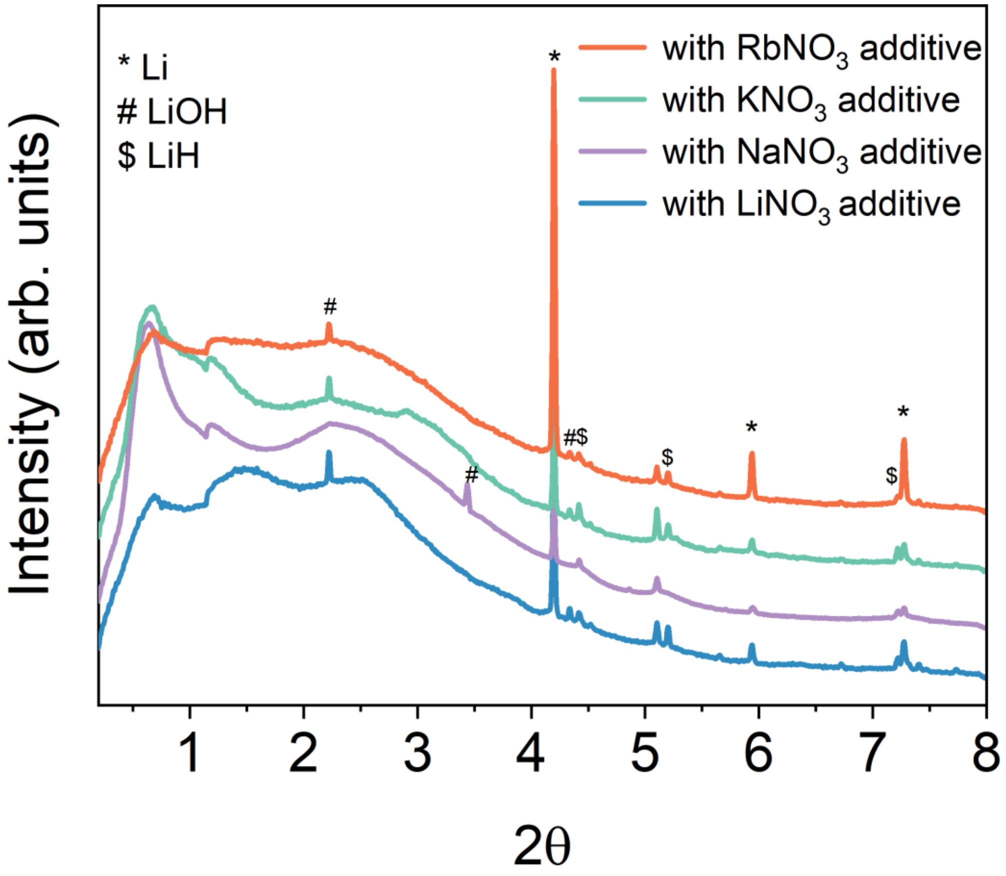

Figure S5. XRD characterization of the SEI components of the Li metal cycled in electrolyte containing $LiNO_3$, $NaNO_3$, KNO_3, and $RbNO_3$ additives.

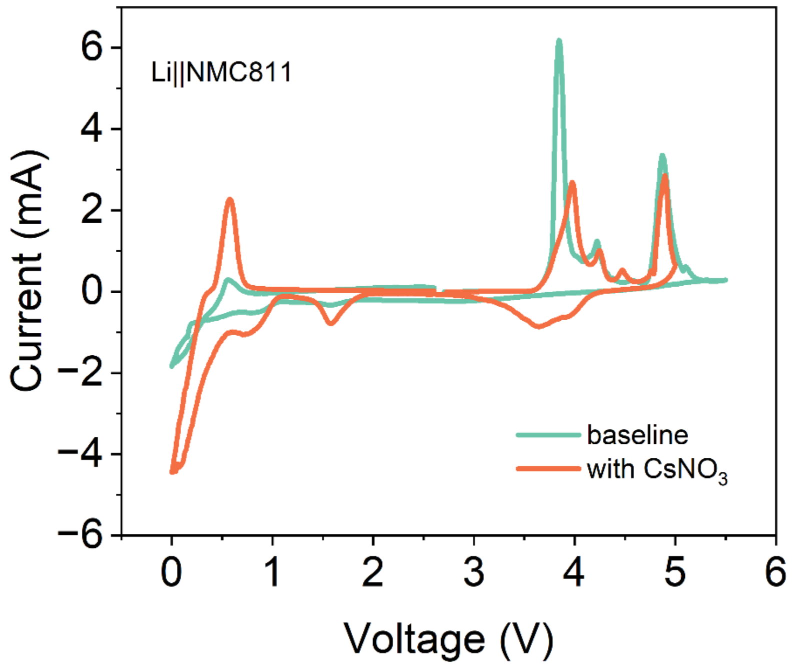

Figure S6. Cyclic voltammetry curves of Li||NMC811 cells cycled in 1.5 M LiFSI in DME (baseline) electrolyte and electrolyte with $CsNO_3$ additive.

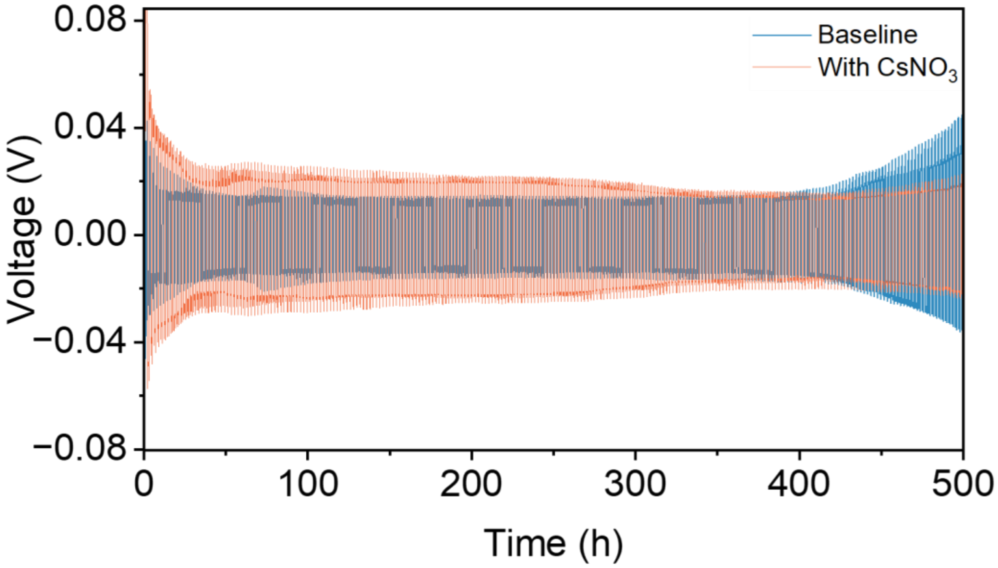

Figure S7. Li||Li symmetric cell cycling performance of baseline electrolyte and electrolyte with 3 wt% $CsNO_3$ additive.

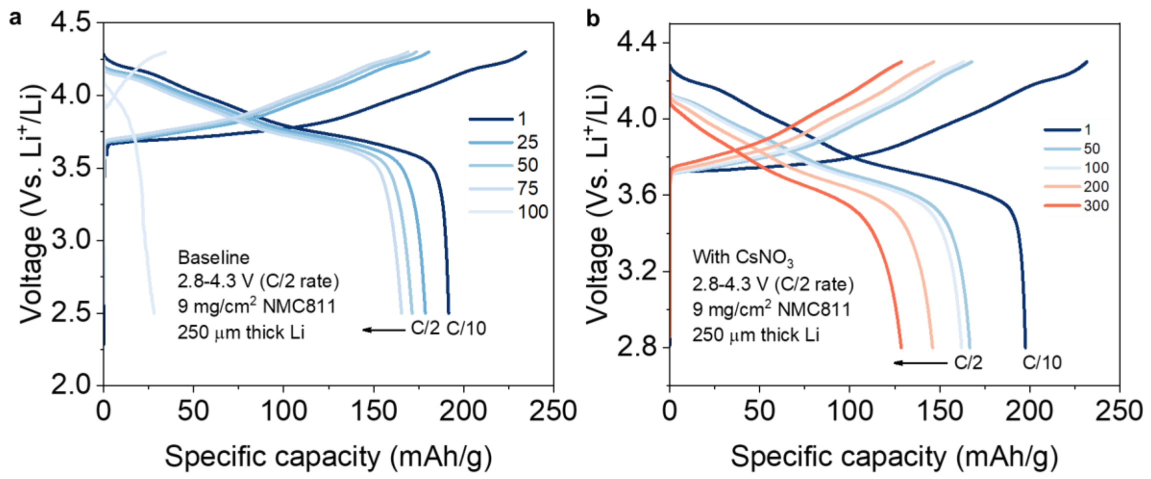

Figure S8. Charge-discharge curves of Li||NMC811 cell with (a) baseline electrolyte, and (b) electrolyte with $CsNO_3$ additive at high N/P cycling condition. Cycling is performed at C/2 rate (0.8 mA/cm^2) after two formation cycles at C/10 rate (0.16 mA/cm^2).

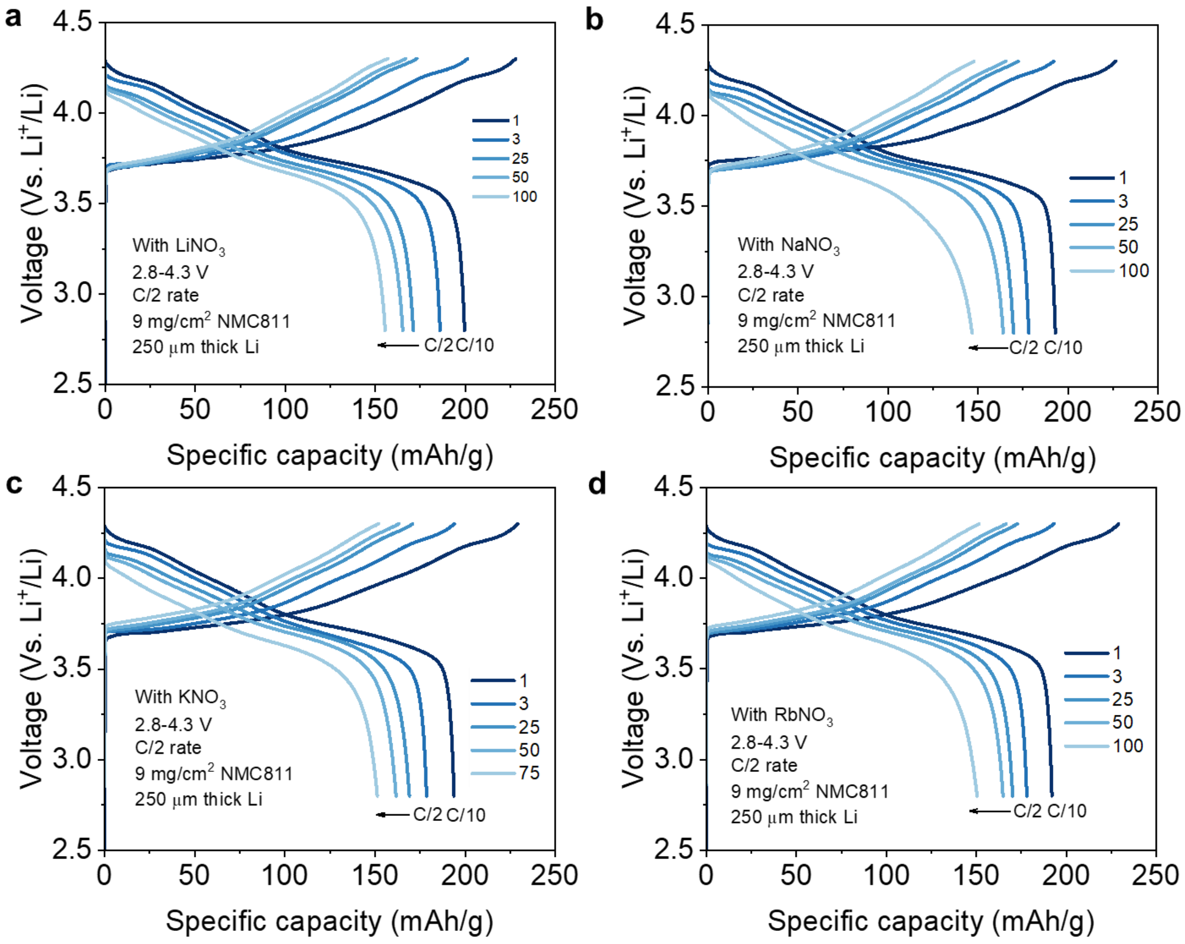

Figure S9. Charge-discharge curves of Li||NMC811 cells with electrolytes containing the (a) $LiNO_3$, (b) $NaNO_3$, (c) KNO_3, and (d) $RbNO_3$ additives at high N/P conditions. Cycling is performed at C/2 rate (0.8 mA/cm^2). Formation cycles were performed at C/10 rate (0.16 mA/cm^2) for 2 cycles.

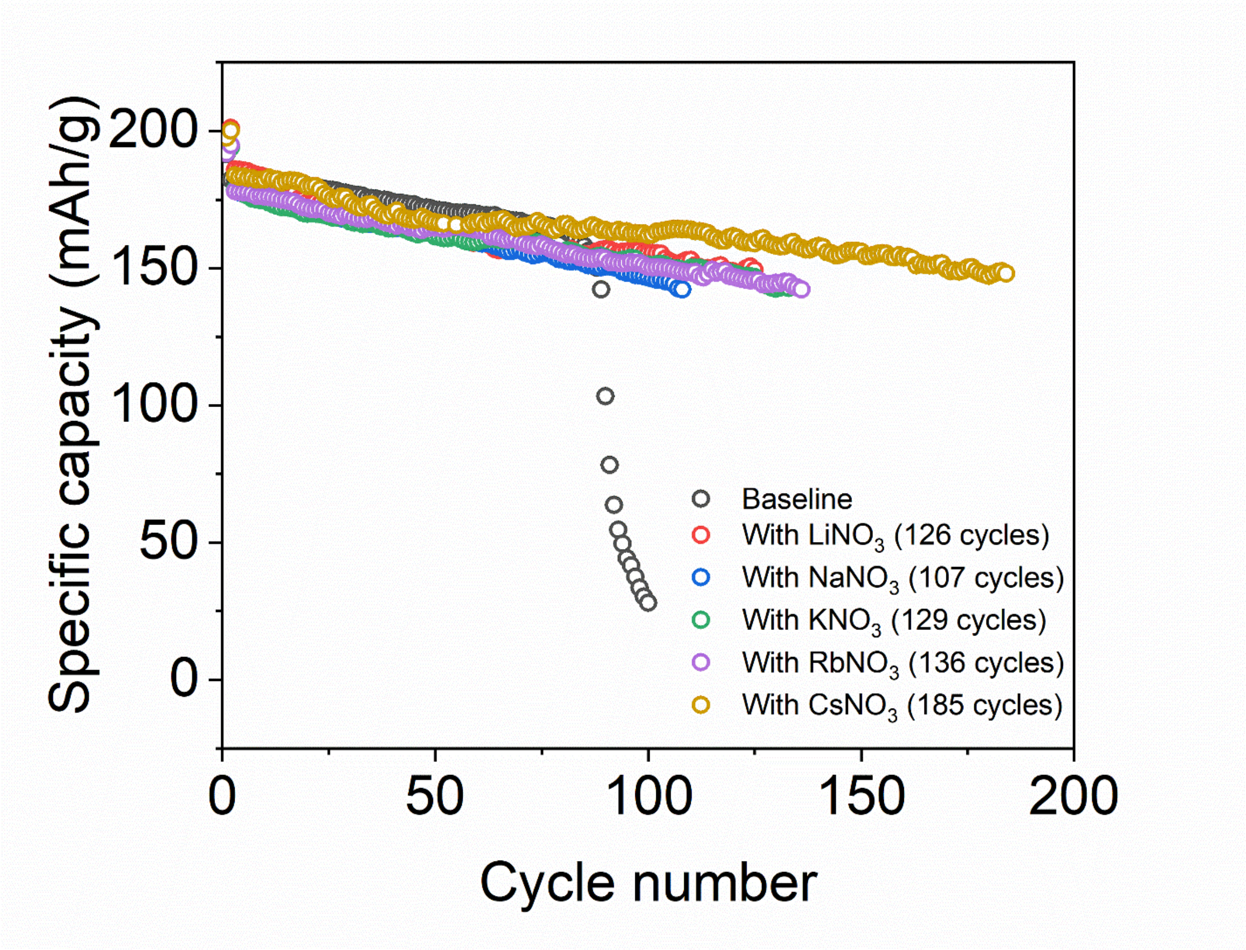

Figure S10. Cyclability of Li metal batteries in electrolytes with different additives. 250 μm thick Li metal is used along with 9 mg/cm^2 loading NMC811 for cycling. Additives utilized are 3 wt% $LiNO_3$, 2 wt% $NaNO_3$, 3 wt% KNO_3, 3 wt% $RbNO_3$, and 3 wt% $CsNO_3$ in baseline electrolyte. Data is shown up to the cycle numbers where the capacity retention reaches 80%.

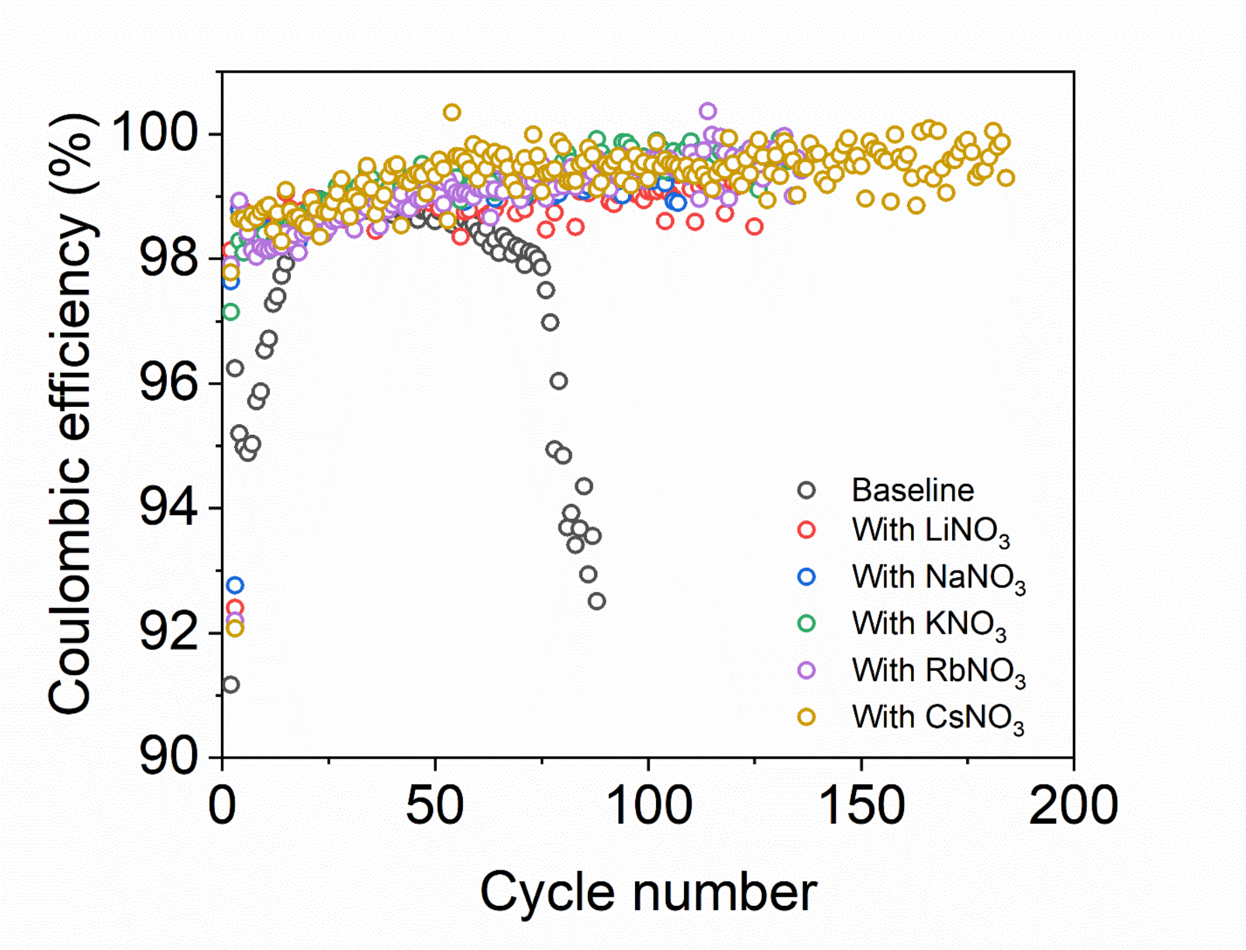

Figure S11. Coulombic efficiency of Li metal batteries with 250 μm thick Li metal and NMC811 (9 mg/cm^2) cycled in baseline electrolyte and electrolyte with different alkali metal nitrate additives.

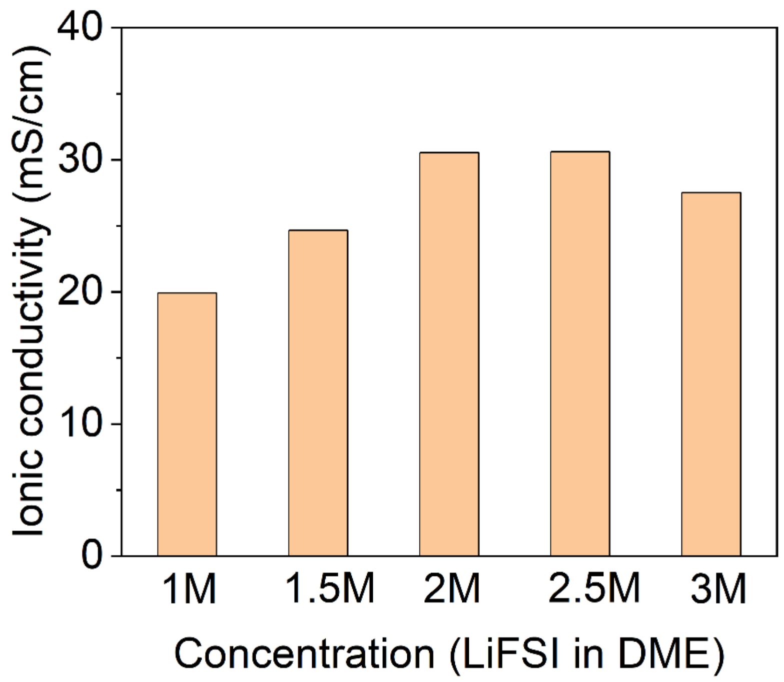

Figure S12. Concentration vs ionic conductivity plot of various LiFSI in DME electrolytes. The ionic conductivities are measured at room temperature.

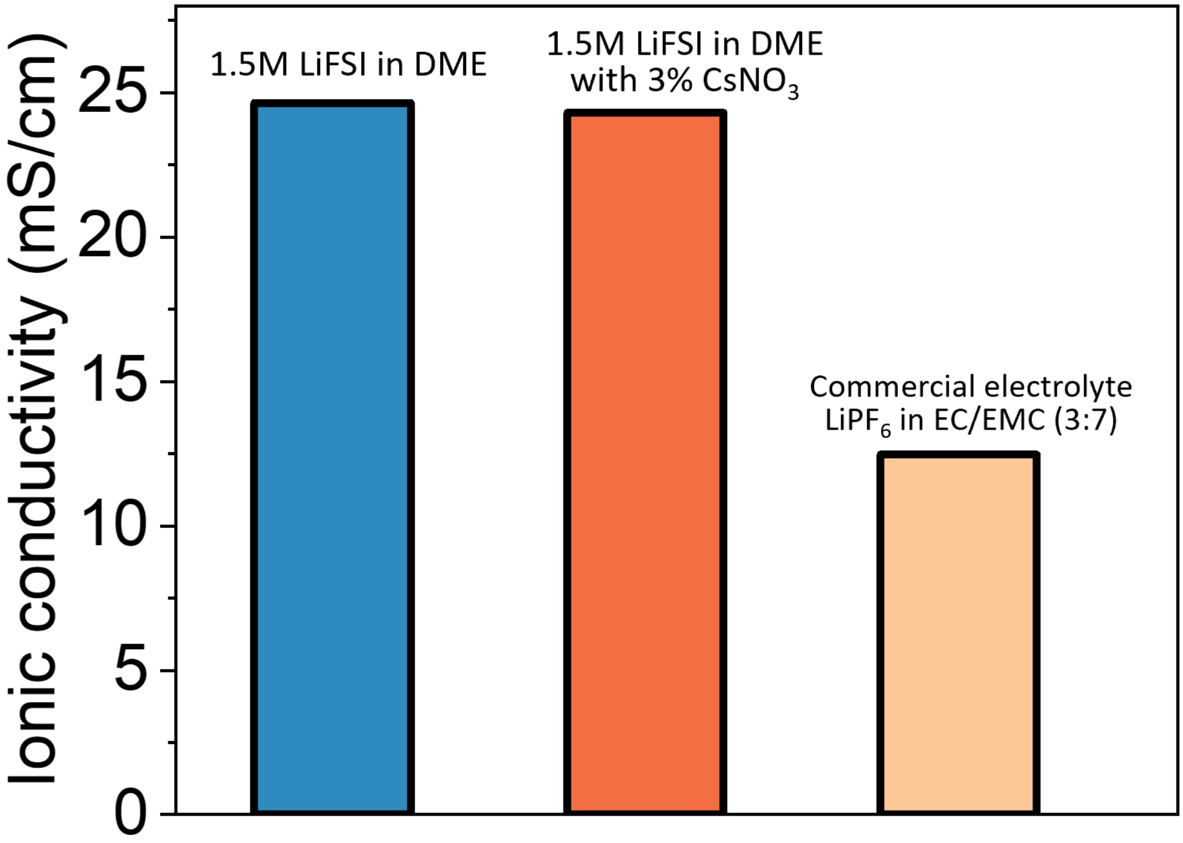

Figure S13. Ionic conductivity comparison of LiFSI in DME electrolyte without and with the $CsNO_3$ additive and commercial carbonate-based electrolyte. The ionic conductivities are measured at room temperature.

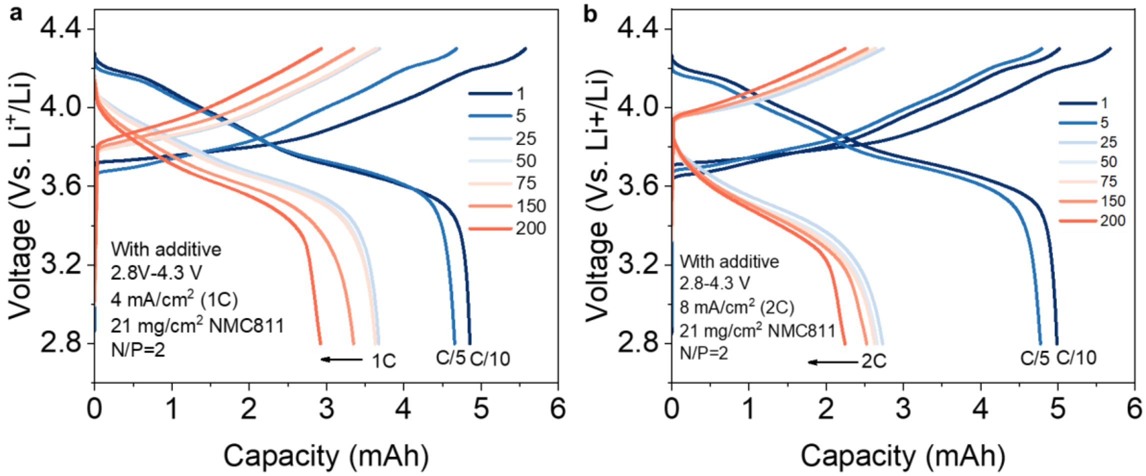

Figure S14. Charge-discharge curves of Li||NMC811 cell with electrolyte containing the $CsNO_3$ additive at (a) 1C (4 mA/cm^2), and (b) 2C rates (8 mA/cm^2) at low N/P cycling conditions. The batteries are cycled at these rates after two formation cycles at C/10 rate (0.4 mA/cm^2) and 5 cycles at C/5 rate (0.8 mA/cm^2).

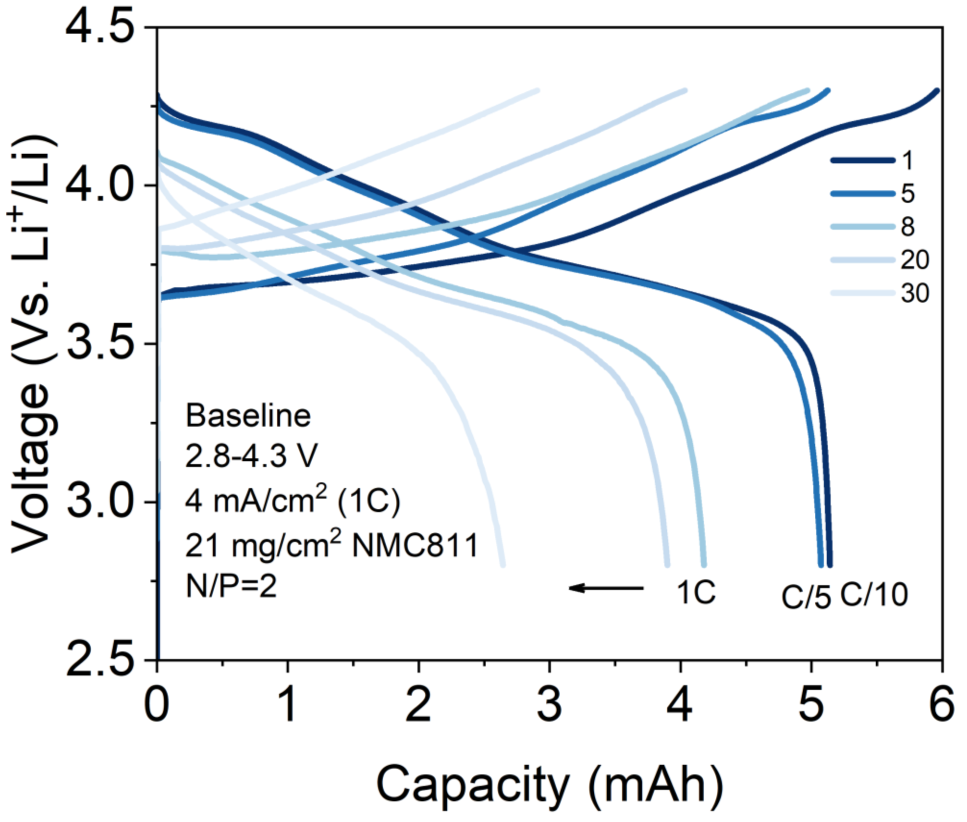

Figure S15. Charge-discharge curves of Li||NMC811 cell cycled at 1C rate (4 mA/cm^2) in the baseline electrolyte at low N/P condition. Formation cycles are performed at C/10 rate (0.4 mA/cm^2) for 2 cycles and 5 cycles at C/5 rate (0.8 mA/cm^2).

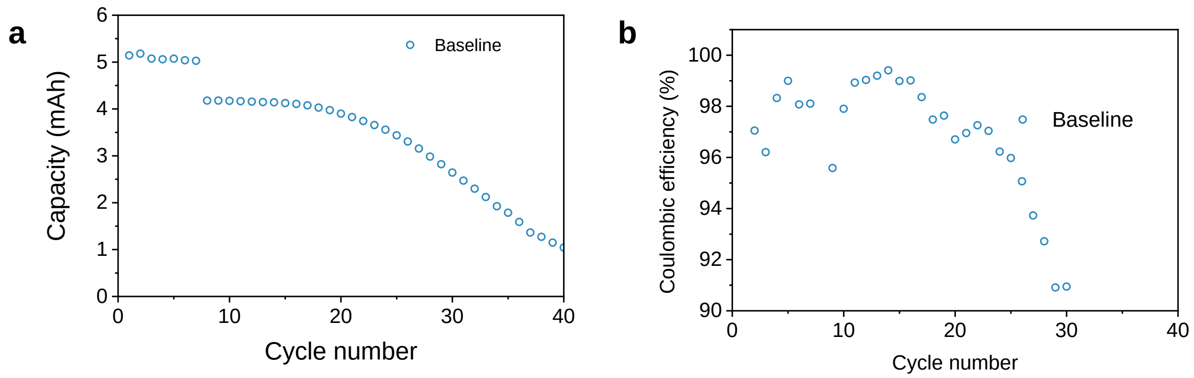

Figure S16. (a) Cycling performance and (b) Coulombic efficiency of Li||NMC811 cell using the baseline electrolyte, 21 mg/cm^2 loading NMC811, and 50 μm thick Li metal at 4mA/cm^2 cycling current density (1C).

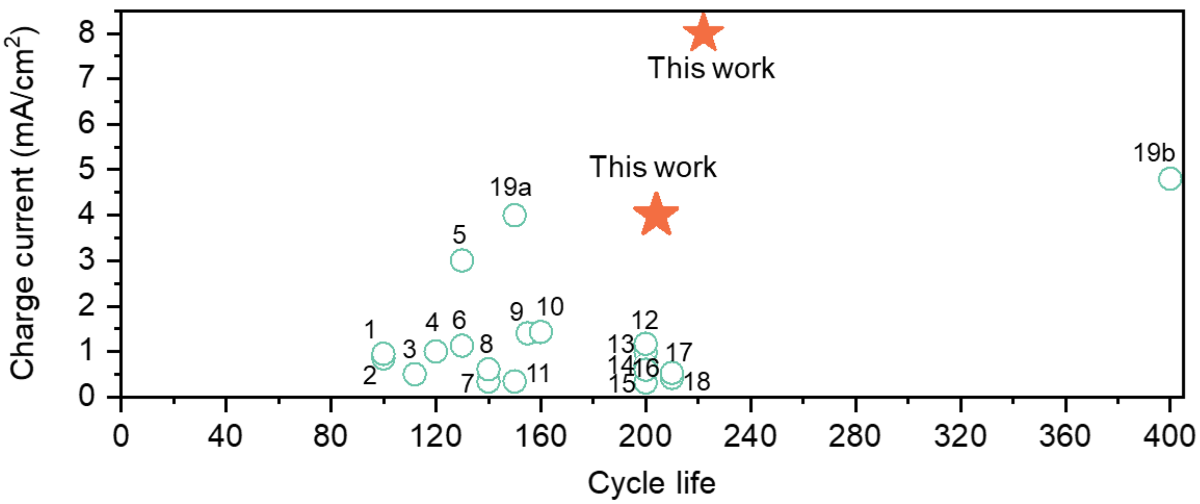

Figure S17. Comparison of literature reported charge current density and cycle life of LMB with this work.[1-19] Data points for ref. 19 utilize two different positive electrode loadings. 19a: 20 mg/cm^2 and 19b: 8 mg/cm^2.

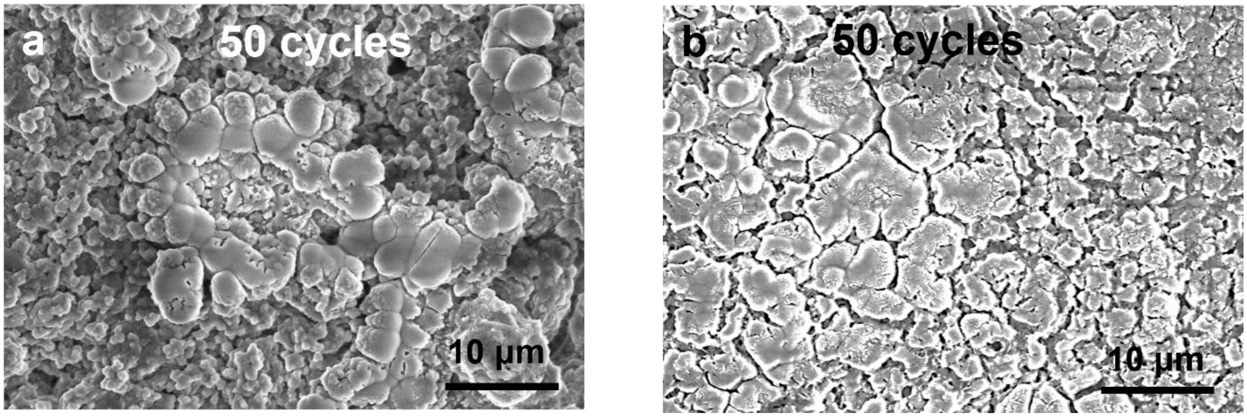

Figure S18. Li metal deposition morphology after 50 cycles in baseline electrolyte (a) and in electrolyte with $CsNO_3$ additive (b).

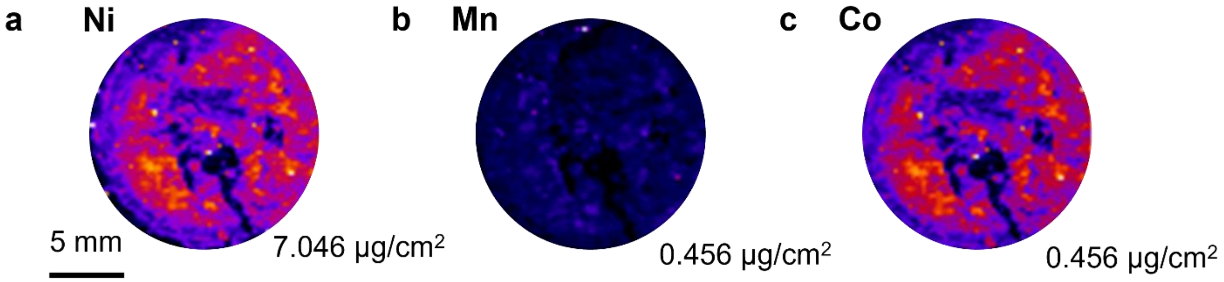

Figure S19. X-ray fluorescence imaging of (a) Ni, (b) Mn, and (c) Co on Li metal cycled in electrolyte containing $CsNO_3$ additive for 200 cycles.

Table S1. Crystallographic data obtained by the Rietveld refinement of the XRD pattern in the top panel of Figure 2a.

**atom**	**site**	**x**	**y**	**z**	**occupancy**	**B value**
**Li space group: $Im\bar{3}m$ a=b=c=3.513(1) Å phase fraction: 31.09%**						
Li	2a	0	0	0	1	3
**LiF space group: $Fm\bar{3}m$ a=b=c=4.05(1) Å phase fraction: 54.90%**						
Li	4a	0	0	0	1	2.484
F	4b	0.5	0.5	0.5	1	3
**LiH space group: $Fm\bar{3}m$ a=b=c=4.084(1) Å phase fraction: 3.34%**						
Li	4a	0	0	0	1	2.0
H	4b	0.5	0.5	0.5	1	2.0
**Li_2O space group: $Fm\bar{3}m$ a=b=c=4.553(1) Å phase fraction: 10.66%**						
O	4a	0	0	0	1	0.3
Li	8c	0.25	0.25	0.25	1	0.2

Table S2. Crystallographic data obtained by the Rietveld refinement of the XRD pattern in the bottom panel of Figure 2a.

**atom**	**site**	**x**	**y**	**z**	**occupancy**	**B value**
**Li space group: Im$\bar{3}$m a=b=c=3.509(1) Å phase fraction: 68.92%**						
Li	2a	0	0	0	1	4.18
**cis-CsFSI space group: P21/n a=7.747(1) Å, b=8.734(1) Å, c=10.088(1) Å phase fraction: 18.37%**						
Cs1		0.287	0.155	0.395	1	2.82
N1		0.651	0.353	0.379	1	0.2
S1		0.746	0.336	0.514	1	4.6
S2		0.691	0.483	0.277	1	4.6
O1		0.754	0.472	0.591	1	3.59
O2		0.682	0.203	0.578	1	3.59
O3		0.869	0.479	0.233	1	3.59
O4		0.575	0.469	0.171	1	3.59
F1		0.935	0.302	0.476	1	3.79
F2		0.664	0.633	0.345	1	3.79
**trans-CsFSI space group: P21/n a=7.899(1) Å, b=8.472(1) Å, c=10.678(1) Å phase fraction: 1.8%**						
Cs1		0.150	0.254	0.413	1	7.5
N1		0.562	0.299	0.444	1	8.0
S1		0.636	0.399	0.342	1	8.0
S2		0.683	0.192	0.54	1	8.0
O1		0.503	0.496	0.279	1	0.2
O2		0.800	0.466	0.386	1	0.2
O3		0.808	0.106	0.488	1	0.2
O4		0.584	0.114	0.619	1	0.2
F1		0.670	0.277	0.241	1	8.0
F2		0.790	0.315	0.630	1	8.0
**LiH space group: Fm$\bar{3}$m a=b=c=4.084(1) Å phase fraction: 6.58%**						
Li	4a	0	0	0	1	0.85
H	4b	0.5	0.5	0.5	1	2.0
**LiOH space group: P4/nmm:2 a=b=3.43(1) Å, c=4.71(1) Å phase fraction: 4.33%**						
O	2c	0.25	0.25	0.184	1	5
Li	2a	0.75	0.25	0	1	0.1
H	2c	0.25	0.25	0.41	1	2

Table S3. Crystallographic data obtained by the Rietveld refinement of the XRD pattern of the SEI species after 100 cycles. Refined XRD pattern is shown in Figure S3.

**atom**	**site**	**x**	**y**	**z**	**occupancy**	**B value**
**Li space group: $Im\bar{3}m$ a=b=c=3.511(1) Å phase fraction: 61.63%**						
Li	2a	0	0	0	1	4.54
**cis-CsFSI space group: P21/n a=7.753(1) Å, b=8.738(1) Å, c=10.085(1) Å phase fraction: 5.1%**						
Cs1		0.287	0.155	0.395	1	4.03
N1		0.651	0.353	0.379	1	0.2
S1		0.746	0.336	0.514	1	4.83
S2		0.691	0.483	0.277	1	4.83
O1		0.754	0.472	0.591	1	2.49
O2		0.682	0.203	0.578	1	2.49
O3		0.869	0.479	0.233	1	2.49
O4		0.575	0.469	0.171	1	2.49
F1		0.935	0.302	0.476	1	0.2
F2		0.664	0.633	0.345	1	0.2
**trans-CsFSI space group: P21/n a=7.898(1) Å, b=8.469(1) Å, c=10.679(1) Å phase fraction: 9.38%**						
Cs1		0.150	0.254	0.413	1	3.53
N1		0.562	0.299	0.444	1	0.2
S1		0.636	0.399	0.342	1	5.14
S2		0.683	0.192	0.54	1	5.14
O1		0.503	0.496	0.279	1	2.39
O2		0.800	0.466	0.386	1	2.39
O3		0.808	0.106	0.488	1	2.39
O4		0.584	0.114	0.619	1	2.39
F1		0.670	0.277	0.241	1	0.2
F2		0.790	0.315	0.630	1	0.2
**LiH space group: $Fm\bar{3}m$ a=b=c=4.084(1) Å phase fraction: 10.67%**						
Li	4a	0	0	0	1	1.77
H	4b	0.5	0.5	0.5	1	2.0
**LiOH space group: P4/nmm:2 a=b=3.55(1) Å, c=4.43(1) Å phase fraction: 13.21%**						
O	2c	0.25	0.25	0.179	1	4.937
Li	2a	0.75	0.25	0	1	0.407
H	2c	0.25	0.25	0.41	1	2

Table S4. Comparison of cycling performance of Li-metal batteries between some of the state-of-the-art electrolytes and the electrolyte reported in this work.

Electrolyte	Electrode types and N/P	Charging current	Cycle life	Reference
3.2 mol/kg LiFSI in C3mpyrFSI-DME in 80:20 weight ratio	NMC622‖Li N/P=5.26	0.95 mA/cm^2	94% retention after 100 cycles	1
1M LiFSI in DMTMSA	NMC811‖Li N/P=7.06	0.85 mA/cm^2	88% retention after 100 cycles	2
2M LiFSI in CFTOF	NMC811‖Li N/P=4	0.5 mA/cm^2	84% retention after 112 cycles	3
1M LiPF6 in FEC-FEMC-HFE	NMC811‖Li N/P=1	1 mA/cm^2	95% retention after 120 cycles	4
2M LiFSI in DME-BTFE	NMC811‖Li N/P=0.92	3 mA/cm^2	80% retention after 130 cycles	5
1M $LiPF_6$ in EC-DEC with 0.5 wt% $Sn(OTf)_2$ and 5 wt% $LiNO_3$	NMC811‖Li N/P=2.65	1.13 mA/cm^2	89.6% retention after 130 cycles	6
1M $LiPF_6$ in FEC-EMC with 3 wt% $LiNO_3$ and 1 wt% TPFPB	NMC811‖Li N/P=2.94	0.34 mA/cm^2	80% retention after 140 cycles	7
7M LiFSI in FEC	LNMO‖Li N/P=1.37	0.61 mA/cm^2	70% retention after 140 cycles	8
1 LiFSI-1.2 DME-3 TTE	NMC811‖Li N/P=2.38	1.4 mA/cm^2	80% retention after 155 cycles	9
1M $LiPF_6$ in EC-DEC with 10 mM $In(OTf)_3$ and 0.5 M $LiNO_3$	NMC811‖Li N/P=2.33	1.43 mA/cm^2	80% retention after 160 cycles	10
1M $LiPF_6$ in FEC-BTC (30:70 ratio by volume)	NMC811‖Li N/P=5.88	0.34 mA/cm^2	89.2% retention after 150 cycles	11
1.5 M LiFSI in DMMS	NMC811‖Li N/P=1.1	1.17 mA/cm^2	88% retention after 200 cycles	12
1.2 M LiFSI in F5DEE	NMC811‖Li N/P=2.04	0.98 mA/cm^2	80% retention after 200 cycles	13
1M LiFSI in DME-BTFE (20:80 by volume)	NMC811‖Li N/P=2	0.7 mA/cm^2	91.4% retention after 200 cycles	14
1M LiFSI in FSA	NMC622‖Li N/P=7.5	0.32 mA/cm^2	89% retention after 200 cycles	15
1M LiFSI in Me_2O-TEE-PFE	NMC622‖Li N/P=111	0.6 mA/cm^2	90.4% retention after 200 cycles	16
1M LiFSI in FDMB	NMC532‖Li N/P=2.5	0.53 mA/cm^2	100% retention after 210 cycles	17
1M LiFSI in 1.3DME-2TFEO	NMC811‖Li N/P=2.38	0.42 mA/cm^2	80% retention after 210 cycles	18

2M LiFSI in TFDPM	NMC811‖Li N/P=1	4 mA/cm^2	88% retention after 150 cycles	19
2M LiFSI in TFDMP	NMC811‖Li N/P=2.5	4.8 mA/cm^2	93% retention after 400 cycles	19
1.5 M LiFSI in DME with 3wt% $CsNO_3$	NMC811‖Li N/P=2	4 mA/cm^2	80% retention after 204 cycles	This work
1.5 M LiFSI in DME with 3wt% $CsNO_3$	NMC811‖Li N/P=2	8 mA/cm^2	80% retention after 224 cycles	This work

References

1 Pal, U. *et al.* Interphase control for high performance lithium metal batteries using ether aided ionic liquid electrolyte. *Energy Environ. Sci.* **15**, 1907-1919 (2022).

2 Xue, W. *et al.* Ultra-high-voltage Ni-rich layered cathodes in practical Li metal batteries enabled by a sulfonamide-based electrolyte. *Nat. Energy* **6**, 495-505 (2021).

3 Zhou, T., Zhao, Y., El Kazzi, M., Choi, J. W. & Coskun, A. Integrated ring-chain design of a new fluorinated ether solvent for high-voltage lithium-metal batteries. *Angew. Chem. Int. Ed.* **61**, e202115884 (2022).

4 Fan, X. *et al.* Non-flammable electrolyte enables Li-metal batteries with aggressive cathode chemistries. *Nat. Nanotechnol.* **13**, 715-722 (2018).

5 Wu, Z. *et al.* Growing single-crystalline seeds on lithiophobic substrates to enable fast-charging lithium-metal batteries. *Nat. Energy* **8**, 340-350 (2023).

6 Zhang, W. *et al.* Colossal granular lithium deposits enabled by the grain-coarsening effect for high-efficiency lithium metal full batteries. *Adv. Mater.* **32**, 2001740 (2020).

7 Li, S. *et al.* Synergistic dual-additive electrolyte enables practical lithium-metal batteries. *Angew. Chem. Int. Ed.* **59**, 14935-14941 (2020).

8 Suo, L. *et al.* Fluorine-donating electrolytes enable highly reversible 5-V-class Li metal batteries. *Proc. Natl. Acad. Sci.* **115**, 1156-1161 (2018).

9 Ren, X. *et al.* Enabling high-voltage lithium-metal batteries under practical conditions. *Joule* **3**, 1662-1676 (2019).

10 Zhang, W. *et al.* Engineering Wavy-Nanostructured Anode Interphases with Fast Ion Transfer Kinetics: Toward Practical Li-Metal Full Batteries. *Adv. Funct. Mater.* **30**, 2003800 (2020).

11 Xiao, P. *et al.* A nonflammable electrolyte for ultrahigh-voltage (4.8 V-class) Li||NCM811 cells with a wide temperature range of 100 C. *Energy Environ. Sci.* **15**, 2435-2444 (2022).

12 Huang, Y. *et al.* Eco-friendly electrolytes via a robust bond design for high-energy Li metal batteries. *Energy Environ. Sci.* **15**, 4349-4361 (2022).

13 Yu, Z. *et al.* Rational solvent molecule tuning for high-performance lithium metal battery electrolytes. *Nat. Energy* **7**, 94-106 (2022).

14 Holoubek, J. *et al.* Electrolyte design implications of ion-pairing in low-temperature Li metal batteries. *Energy Environ. Sci.* **15**, 1647-1658 (2022).

15 Xue, W. *et al.* FSI-inspired solvent and “full fluorosulfonyl” electrolyte for 4 V class lithium-metal batteries. *Energy Environ. Sci.* **13**, 212-220 (2020).

16 Yin, Y. *et al.* Fire-extinguishing, recyclable liquefied gas electrolytes for temperature-resilient lithium-metal batteries. *Nat. Energy* **7**, 548-559 (2022).

17 Yu, Z. *et al.* Molecular design for electrolyte solvents enabling energy-dense and long-cycling lithium metal batteries. *Nat. Energy* **5**, 526-533 (2020).

18 Cao, X. *et al.* Optimization of fluorinated orthoformate based electrolytes for practical high-voltage lithium metal batteries. *Energy Storage Mater.* **34**, 76-84 (2021).
19 Zhao, Y., Zhou, T., Mensi, M., Choi, J. W. & Coskun, A. Electrolyte engineering via ether solvent fluorination for developing stable non-aqueous lithium metal batteries. *Nat. Commun.* **14**, 299 (2023).
